# Assessing the Efficacy and Safety of Extubation Protocols in the Intensive Care Unit Following Transoral Robotic Surgery for Obstructive Sleep Apnea Syndrome: A Retrospective Cohort Study

**DOI:** 10.3390/jcm13226786

**Published:** 2024-11-11

**Authors:** Andreaserena Recchia, Marco Cascella, Massimiliano Copetti, Alessio Barile, Elena Giovanna Bignami, Aurelio D’Ecclesia, Antonio Izzi, Aldo Manuali, Vincenzo Marchello, Giuseppe Mincolelli, Alfredo Del Gaudio

**Affiliations:** 1 Anesthesia e Intensive Care 2, IRCCS “Casa Sollievo della Sofferenza” San Govanni Rotondo, 7103 Foggia, Italy; a.barile@operapadrepio.it (A.B.); a.izzi@operapadrepio.it (A.I.); a.manuali@operapadrepio.it (A.M.); v.marchello@operapadrepio.it (V.M.); g.mincolelli@operapadrepio.it (G.M.); 2 Anesthesia and Pain Medicine, Department of Medicine, Surgery and Dentistry “Scuola Medica Salernitana”, University of Salerno, Baronissi, 84081 Salerno, Italy; 3 Unit of Biostatics, IRCCS “Casa Sollievo della Soffrenza” San Giovanni Rotondo, 7103 Foggia, Italy; m.copetti@operapadrepio.it; 4 Anesthesiology, Critical Care and Pain Medicine Division, Department of Medicine and Surgery, University of Parma, 43126 Parma, Italy; elenagiovanna.bignami@unipr.it; 5 ENT Operative Unit, IRCCS “Casa Sollievo della Soffrenza” San Giovanni Rotondo, 7103 Foggia, Italy; a.decclesia@operapadrepio.it; 6 Past Director Anesthesia e Intensive Care 2, IRCCS “Casa Sollievo della Soffrenza” San Giovanni Rotondo, 7103 Foggia, Italy; freddydelgaudio@libero.it

**Keywords:** obstructive sleep apnea syndrome, transoral robotic surgery, safety, anesthesia

## Abstract

**Background**: There is a notable lack of protocols addressing extubation techniques in transoral robotic surgery (TORS) for obstructive sleep apnea (OSA). **Methods**: This retrospective cohort study enrolled patients who underwent TORS for OSA between March 2015 and December 2021 and were managed with different extubation approaches. The patients were divided into two groups: high-flow nasal cannula (HFNC) therapy and conventional oxygen therapy. The use of an airway exchange catheter (AEC) was investigated. **Results**: The application of HFNC use versus conventional oxygen therapy led only to a statistical reduction in extubation time (*p* = 0.024); length of stay in the intensive care unit (ICU) and the episodes of desaturation below 95% were reduced, but data are non-statistically significant. Similarly, the application of an AEC led to a reduction in extubation time in hours (*p* = 0.008) and length of stay in the ICU (*p* = 0.024). **Conclusions**: In patients with OSA who underwent TORS, the use of an HFNC, with or without an AEC, resulted in a significant reduction in extubation time without major adverse events. Additionally, HFNC utilization may decrease desaturation episodes during extubation. Despite limitations, based on the findings of this preliminary investigation, the combination of an HFNC and an AEC emerges as a promising strategy for enhancing the safety and efficacy of extubation protocols in this patient population.

## 1. Introduction

Obstructive sleep apnea (OSA) is a prevalent condition affecting between 2% and 34% of adults. It is linked to cardiovascular disorders, cognitive dysfunction, strokes, and increased mortality rates [1,2,3,4]. The current standard therapy for moderate to severe OSA patients involves continuous positive airway pressure (CPAP) [5,6]. Depending on the severity and unique features of the condition, alternative therapeutic options are available. These encompass lifestyle modifications, dietary adjustments, positional therapy, oral appliances, and surgical interventions, which can also be combined for a more comprehensive approach [7,8]. Transoral robotic surgery (TORS) has emerged as a minimally invasive surgical approach with promising outcomes. This approach is utilized for patients experiencing obstruction of the airway due to hypertrophy of the lingual tonsils. In such cases, the base of the tongue is reduced, resulting in a broader airway and reducing the likelihood of collapse [9].

Post-surgical patients, under general anesthesia and ventilation via an endotracheal tube, necessitate careful management during the awakening and extubation process, typically in an intensive care setting. Extubation is an elective procedure requiring meticulous evaluation and planning, particularly when difficult airway management is anticipated. Complications associated with extubation can lead to increased morbidity and mortality [10,11].

The literature highlights common complications following TORS, including tongue edema, tongue and pharyngeal paresthesia, minor and major hemorrhage, dysgeusia, dysphagia with an increased risk of aspiration, and pain. Various devices are suggested to enhance the safety of difficult extubation [9]. The airway exchange catheter (AEC), for example, proves useful for potential reintubation scenarios, where it serves as a guide for tube reinsertion [12]. Additionally, a high-flow nasal cannula (HFNC) oxygen-delivery approach improves ventilation post-extubation, reducing respiratory insufficiency. HFNC application diminishes nasopharyngeal dead space, increases alveolar oxygen fraction, lessens respiratory muscle fatigue, improves secretion management, and decreases upper airway obstruction incidents [13,14]. These two devices are complementary but non-equal in managing difficult airways. While the AEC is a useful guide for reintubation in difficult airways, it does not contribute to oxygenation and ventilation unlike an HFNC.

Despite the existing literature, there is a notable absence of protocols addressing extubation techniques in the intensive care unit (ICU) following TORS. This retrospective analysis aims to assess the efficacy of different protocols, shedding light on their impact on patient outcomes and complications in this specific clinical context.

## 2. Materials and Methods

### 2.1. Study Design and Settings

This study was a single-center retrospective study conducted at the hospital Casa Sollievo della Sofferenza (San Giovanni Rotondo, Foggia, Italy) from March 2015 and December 2021. The study was approved by the local Ethics Committee (N23/CE). This study adhered to ethical standards as outlined in the Declaration of Helsinki and followed the guidelines set by the Strengthening the Reporting of Observational Studies in Epidemiology (STROBE) checklist. All patients gave signed informed consent during preoperative assessment.

The retrospective analysis was conducted by retrieving the cases of patients undergoing TORS for OSA from a register and collecting data in digitalized medical records and subsequent statistical analysis. Patient-related risk factors and surgical techniques were taken into consideration during data collection. The obtained dataset is available at [15].

**Figure 1:**
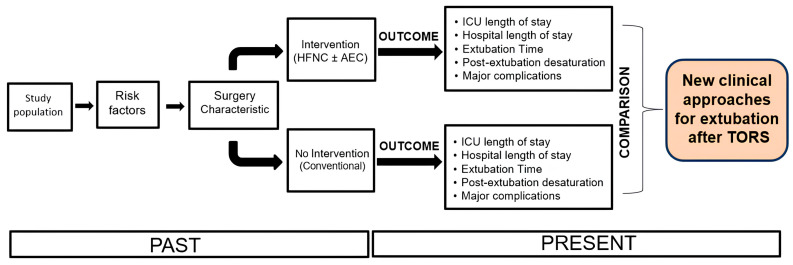
The course of the retrospective study. Abbreviations: airway exchange catheter, AEC; high-flow nasal cannula, HFNC; intensive care unit, ICU.

The favorable outcome was evaluated as a reduction in extubation times, a reduction in ICU and hospital stays, and a reduction in major complications (significant desaturations, major bleeding, and fatal events) (Figure 1).

### 2.2. Study Participants

Patients scheduled for TORS underwent a comprehensive preoperative assessment, which included polysomnography to evaluate their sleep patterns and respiratory function. The surgical procedures were conducted under a remifentanil propofol-based target-controlled infusion (TCI) general anesthesia monitoring the depth of anesthesia. Curarization was administered only at induction, if necessary, as an alternative to laryngotracheal anesthesia with topical lidocaine (LTA). Following the completion of the surgical intervention, patients were transferred to the ICU for postoperative monitoring and management. The decision to awaken and extubate the patients was made only after a thorough multidisciplinary clinical evaluation, which involved collaboration between anesthesiologists and otolaryngologists. This joint assessment aimed to ensure the patient’s readiness for extubation and to mitigate any potential risks or complications associated with the procedure. Patients with incomplete data were excluded from the analysis to maintain the integrity and reliability of the study findings.

### 2.3. Variables

We collected demographics, preoperative use of CPAP, lowest oxygen saturation (SpO2) at preoperative polysomnography, American Society of Anesthesiologists (ASA) physical status, comorbidities, main diagnosis, and Apnea-Hypopnea Index (AHI). The latter is a measure used in sleep medicine to assess the severity of sleep-disordered breathing, particularly in conditions such as OSA. This index quantifies the frequency and severity of apnea (complete cessation of breathing that lasts for at least 10 s) and hypopnea (partial reduction in breathing typically defined as a decrease in airflow of at least 30% accompanied by a reduction in blood oxygen saturation or an arousal from sleep) episodes during sleep. To calculate the AHI, the total number of apneas and hypopneas that occur per hour of sleep is determined. This number is then divided by the total number of hours of sleep to obtain the AHI value. The AHI is considered normal when it is fewer than 5 events per hour; mild sleep apnea is classified when the score falls within the range of 5 to 15 events per hour, moderate from 15 to 30 events per hour, and severe if it exceeds 30 events per hour. A higher AHI indicates a greater severity of sleep-disordered breathing and may be associated with increased health risks, including cardiovascular problems, daytime sleepiness, and impaired cognitive function [16].

Regarding the procedure and postoperative management, we considered the type of surgical TORS, operative times, extubation times, length of hospital and ICU stays, the weaning time from an HFNC, number of episodes of desaturation (SpO2 < 95% and SpO2 < 92%; continuous desaturation was defined as a period during which blood oxygen saturation levels remain consistently below the specific threshold for at least 5 min) in the first 24 h after extubation, the use of an AEC, and hemorrhagic and cardiovascular adverse effects in the first 48 h. For the analyses, we did not consider data on cognitive dysfunctions and/or hemodynamic parameters.

### 2.4. Outcomes

The main expected outcome of the study was to assess whether the application of a different extubation approach was associated with the absence of major complications and a reduction in the time required to remove the endotracheal tube, expressed in hours. Major complications may include events such as infections, lung or organ damage, bleeding, or other complications requiring additional treatment or a prolonged hospital stay.

In addition to the primary outcome, the secondary objective was to assess the duration of stay in the ICU, measured in days, and the number of desaturation episodes, i.e., the reduction in blood oxygen levels below 95%, occurring within the first 24 h after extubation. These episodes may indicate complications or less effective recovery after tube removal and thus can serve as a measure of the safety and efficacy of the extubation protocol.

### 2.5. Statistics

The demographic and clinical characteristics of patients at baseline were reported as mean and standard deviation or as median and range for continuous variables, and as frequency and percentage for categorical variables. Comparisons between groups were conducted using the non-parametric Mann–Whitney test for continuous variables and the exact Fisher test for categorical variables. All analyses were performed using the statistical environment R (4.4.2). A *p*-value < 0.05 was considered statistically significant. The balance analysis between the groups (conventional therapy and HFNC therapy) was performed using propensity score (PS) matching with the key variables age, gender, BMI, ASA, AHI, SpO2, CPAP, and duration of intervention in minutes. Missing data rates in subjects’ covariates at baseline used in this study were, when present, always less than 5%. Missing data, therefore, were imputed using a random forest approach [17].

## 3. Results

**Figure 2:**
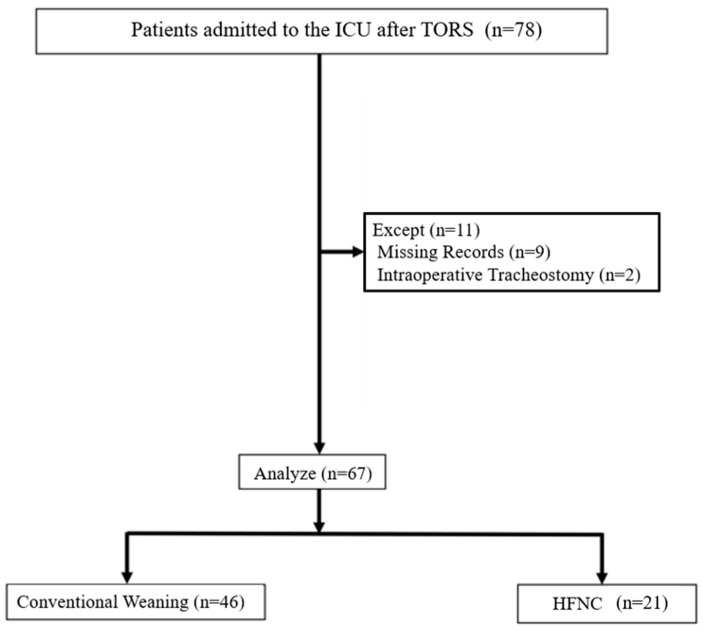
Study flowchart. Abbreviations: TORS, transoral robotic surgery; ICU, intensive care unit; HFNC, high-flow nasal cannula.

A total of 78 patients were initially considered for the analysis. However, 11 cases were excluded due to incomplete data (*n* = 9) or intraoperative tracheostomy (*n* = 2), resulting in a final sample size of 67 patients included in the analysis. In this sample, 46 patients were included in the conventional weaning group and 21 patients in the HFNC group (Figure 2). Both medical devices were used in 18 of 21 patients extubated with an HFNC. Five patients were extubated only with an AEC, and they were included in the conventional extubation group. The small sample did not allow analyzing the outcome separately for two groups.

Among the included cases, 54 patients (80.6%) were male. The mean age of the included patients was 28.3 years (SD = 3.42), with a median age of 28 years. Demographic and preoperative clinical data are reported in Table 1.

Data collected on the perioperative course are reported in Table 2.

### Conventional vs. HFNC management

**Figure 3:**
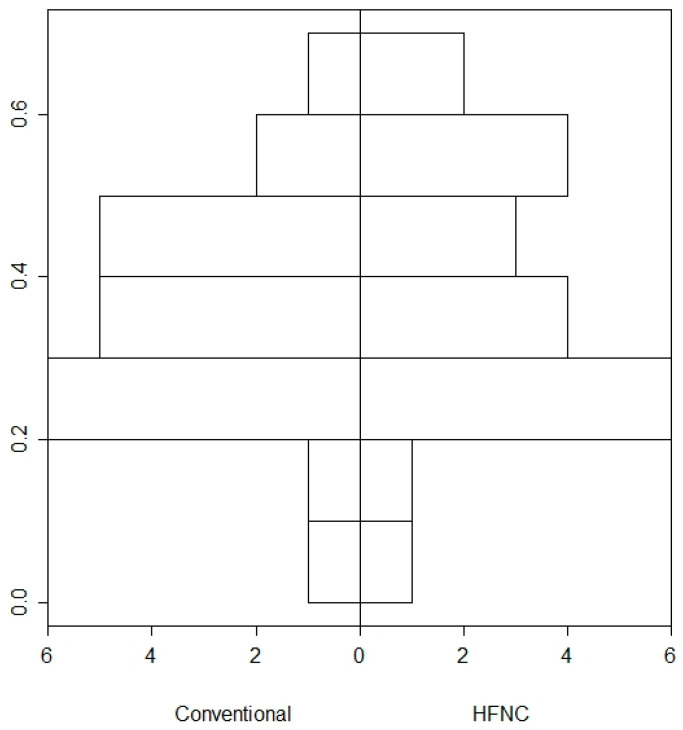
Bar plot of the propensity scores (PSs) distribution between the two groups. The x-axis represents the two groups being compared (Conventional and HFNC). The y-axis represents the density or proportion of patients within specific ranges of PSs. The bars indicate the distribution of propensity scores for each group. Each bar’s height corresponds to the proportion of patients in that group who fall within a specific range of PSs. The alignment of bars between the two groups shows how similar the groups are in terms of their PSs after matching. The graph shows that the PSs of the two groups have been balanced. The bars for the Conventional and HFNC groups should align closely, indicating that the matching process has successfully created comparable groups.

Given the considered variable, a patient-patient matching (*n* = 21) in the Conventional and HFNC groups was performed. The analyses of the PS matching achieved its goal of balancing the two groups. Results from the PS after matching are shown in Figure 3.

In the comparative analysis, involving 46 patients in the Conventional group and 21 patients in the HFNC group, several noteworthy findings emerged. Notably, there was a good homogeneity between groups. There was no statistically significant difference in age (Conventional: 55.3 ± 11.78 years, HFNC: 52.95 ± 11.26 years, *p* = 0.445). Gender distribution was similar in both groups, with approximately 80% male participants in each. Body Mass Index (BMI) also showed no significant difference between groups (Conventional: 28.61 ± 3.59, HFNC: 27.53 ± 2.97, *p* = 0.236). Concerning preoperative characteristics, both groups exhibited comparable AHI scores (Conventional: 25.66 ± 8.76, HFNC: 26.99 ± 10.87, *p* = 0.603). Moreover, preoperative oxygen saturation levels (SpO2) and usage of CPAP were similar across both groups. Furthermore, there were no significant differences in the types of surgeries performed between the groups, and although the duration of surgery was slightly longer in the HFNC group, the difference was not statistically significant (*p* = 0.177) (Table 3).

**Figure 4:**
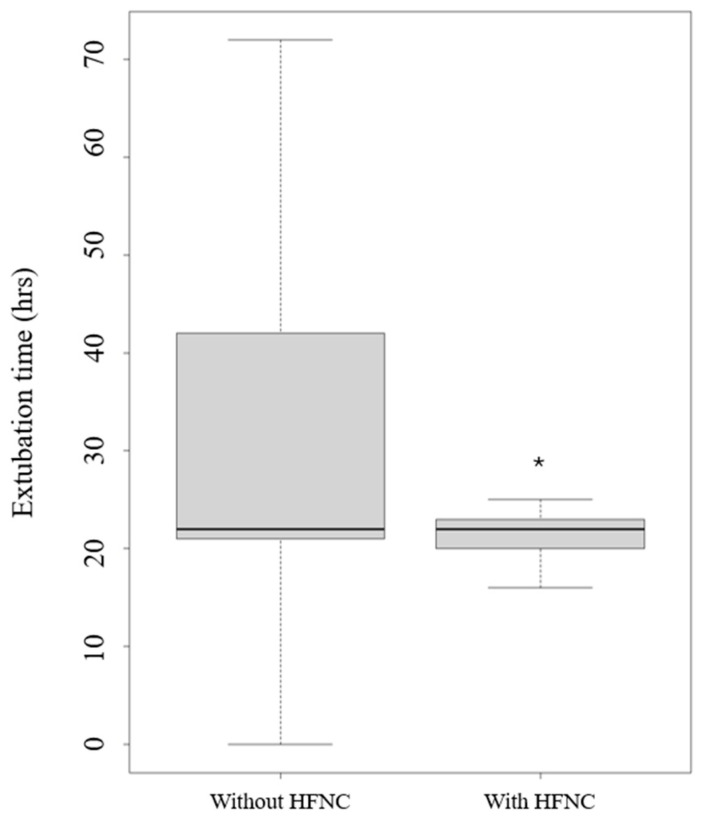
Extubation time with and without high-flow nasal cannula (HFNC). Legend: * *p* < 0.005.

The application of HFNC use versus conventional oxygen therapy led only to a statistical reduction in extubation time (*p* = 0.024) (Figure 4).

Moreover, the length of stay in the ICU and the episodes of desaturation below 95% were reduced, but data are non-statistically significant.

**Figure 5:**
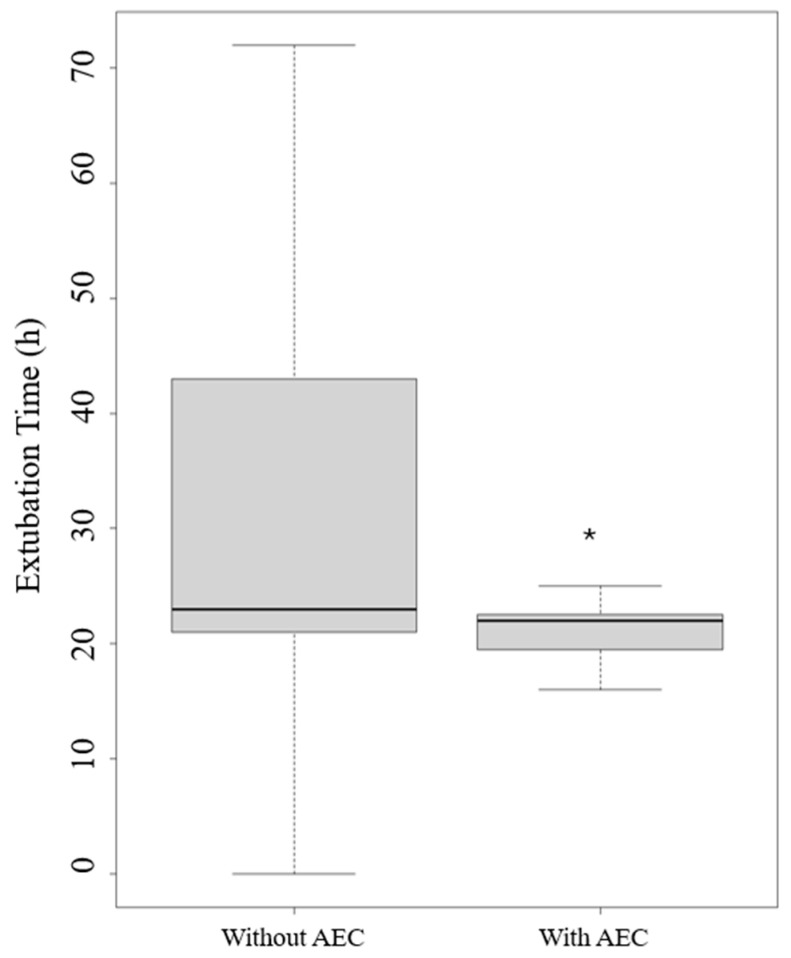
Extubation times with and without an airway exchange catheter (AEC). Legend: * *p* < 0.005.

**Figure 6:**
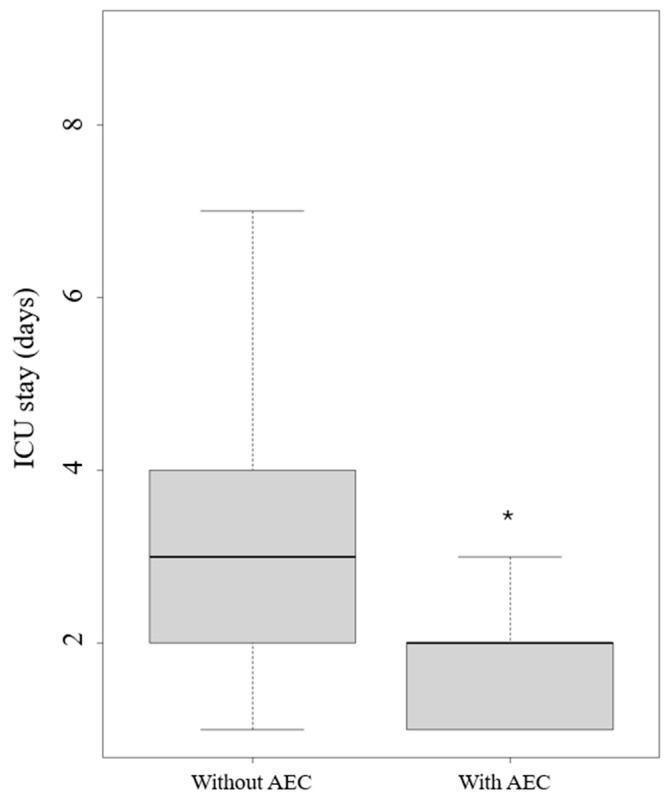
Intensive care unit (ICU) stays with and without an airway exchange catheter (AEC). Legend: * *p* < 0.005.

The application of an AEC led to a reduction in extubation time in hours (*p* = 0.008) (Figure 5) and length of stay in the ICU (*p* = 0.024) (Figure 6).

Regarding significant complications, two patients (0.33%) experienced fatal outcomes in the ICU attributable to hemorrhagic and arrhythmic complications, respectively. Both patients were managed with conventional oxygen therapy after the extubation. No hemorrhagic or cardiovascular adverse effects were collected when a protocol of extubation was applied (HFNC or AEC).

## 4. Discussion

The present study aimed to evaluate the efficacy and safety of extubation protocols following TORS for OSA in patients admitted to the ICU for postoperative care. Our findings suggest that the utilization of an HFNC, with or without an AEC, resulted in a significant reduction in extubation time without major adverse events. Additionally, HFNC application may decrease episodes of desaturation during extubation. These results underscore the potential benefits of incorporating an HFNC and an AEC into extubation protocols in this patient population.

In TORS procedures, the anesthetic approach is typically aimed at minimizing anesthesia recovery duration, reducing postoperative sedation, alleviating severe pain, and mitigating nausea/vomiting [18]. Nevertheless, due to manipulation of the airway and the potential for lengthy surgery, there is a risk of airway edema, bleeding, and blood clots, which could lead to airway complications and aspiration. The ease of securing the airway, the extent of surgical resection, and the patient’s underlying health conditions, such as the severity of obstructive sleep apnea, will determine the level of postoperative monitoring required. Patients who are difficult to intubate, undergo extensive tongue resection, or have high AHI scores may benefit from continued intubation and management in the ICU [19]. Extubation should only occur after full reversal of anesthesia effects, muscle relaxation, control of surgical bleeding, and resolution of swelling. At our center, we routinely reverse and extubate patients in the ICU for optimal postoperative care.

Many studies analyzed the incidence of complications in the postoperative course after TORS with variable results. Recently, Mandloi et al. [20] in a cost analysis study highlighted that the most common complications following TORS include bleeding, dehydration, dysphagia, tongue numbness, and dysgeusia with postoperative bleeding occurring in up to 30% of patients. Additionally, in a systematic revision and metanalysis [21], the mean complication rate for TORS techniques in OSA was 36.7% with 11.9% minor complications, 22.02% moderate, and 2.75% severe (major bleeding at 0.92% and oropharyngeal stenosis at 0.92%). Baptista et al. [22] analyzed a sample of 57 patients admitted to the surgical ICU postoperatively after TORS. They found that the median number of days in the ICU and hospital was shorter than that for our center, as was the mean time of TORS procedure. None of the patients underwent a tracheostomy, but the incidence of major bleeding was higher, with the need for operative intervention in three cases (5.3%). No information on extubation technique was provided. Furthermore, Toppenberg [23] investigated the predictors of success after TORS. No predictors for success were identified if success outcomes were combined. Instead, ASA scores and anticoagulation use were significantly correlated with an increased risk. The mean hospitalization duration was 3.2 days, and hemorrhagic complications occurred in six patients (11.8%). The duration of surgery was shorter, but severe and moderate complications occurred in, respectively, 2% and 3.9% of patients (class IV and III of Clavier Dindo Classification). Although the extubation technique was not analyzed, the authors acknowledged that the occurrence of complications even for benign pathologies implies the need for an extubation strategy.

Therefore, in the future, it would be important to analyze and demonstrate the reduction in mild, moderate, and major complications in the postoperative period also with follow-up compared to the literature.

The application of an extubation strategy has allowed our department to reduce hospitalization and the incidence of major complications. The reduction in extubation time observed with HFNC and AEC utilization aligns with previous studies demonstrating the efficacy of these interventions in improving respiratory outcomes post-extubation, but these studies occur in contexts other than that of TORS. For example, in chronic obstructive pulmonary disease, HFNC therapy has been shown to enhance ventilation, reduce respiratory muscle fatigue, and improve secretion management, thus facilitating the transition from mechanical ventilation to spontaneous breathing [24]. Similarly, the use of an AEC may aid in the smooth removal of the endotracheal tube, particularly in cases where difficult airway management is anticipated [25]. These findings support the notion that combining these interventions can optimize extubation processes and minimize associated complications.

Furthermore, our study highlights the potential role of an HFNC in reducing desaturation episodes during extubation. Desaturation, characterized by a decrease in blood oxygen levels, is a common complication following extubation and can lead to respiratory insufficiency and other adverse outcomes [4,26]. The ability of an HFNC to maintain optimal oxygenation and reduce nasopharyngeal dead space may contribute to the observed reduction in desaturation episodes. However, due to the non-statistically significant results regarding desaturation episodes and length of ICU stay, the findings should be interpreted with caution and underscore the need for further research to elucidate the precise impact of HFNCs on respiratory outcomes in this context.

In our analysis, we calculated that two patients (0.33%) experienced fatal outcomes in the ICU due to hemorrhagic and arrhythmic complications, respectively. However, despite finding that both patients received conventional oxygen therapy after extubation, it is not possible to draw definitive conclusions regarding the link between complications and the weaning technique. There are multiple confounding variables at play, and a large dataset with multivariate analysis would be necessary to elucidate this further. Nonetheless, this surgery is associated with complications, as reported by evidence-based medicine studies. For instance, major hemorrhagic complications can occur in almost 0.9% of procedures [21]. According to the results of the data analysis, in our center, the implemented extubation protocol provides HFNC oxygen administration with an AEC in situ. A multidisciplinary clinical evaluation between surgeons and intensivists is performed for addressing stability of vital parameters, presence of airway protective reflexes, and absence of edema or bleeding at the surgical site.

### Study Limitations

It is worth noting that our study has several limitations. Firstly, its retrospective nature introduces inherent biases and limitations in data collection. Secondly, the small sample size and single-center design may limit the generalizability of our findings, but the limited sample size can play a role in small effect sizes. This issue primarily affects the analyses of key variables. For example, the variable SpO2 < 95 continuous has a mean equal to 1.00 (SD: 1.62) in the Conventional group and 0.52 (1.57) in the HFNC group; therefore, the ex-post power to detect this difference is only 0.20.

Additionally, the lack of a standardized extubation protocol and variations in patient management may have influenced the outcomes assessed.

An important source of bias arises from the non-exclusive utilization of the AEC within a single group. However, the two groups significantly differ in terms of device usage (<0.001; Table 3). Additionally, six patients (8.9%) underwent extubation using both an AEC and conventional oxygen therapy, while three patients (4.4%) were exclusively extubated using HFNC oxygen therapy. This imbalance introduces a significant confounding factor. Due to the relatively small sample size, we were not able to statistically control for AEC unbalance between groups potentially resulting in biased estimates.

Future prospective studies with larger sample sizes and standardized protocols are warranted to validate our findings and further elucidate the optimal approach to extubation in this patient population.

## 5. Conclusions

In patients affected by OSA and admitted to the ICU after TORS, the application of an HFNC with or without an AEC can lead to a significant reduction in extubation time without major adverse events, improving the safety of difficult extubation protocols. According to the results of this pilot study, the use of an HFNC could reduce desaturation episodes during extubation after TORS. Therefore, oxygen therapy through an HFNC combined with an AEC seems to be a safety and efficacy strategy for extubation protocols. Nevertheless, further high-quality research is warranted to confirm these results.

## Figures and Tables

**Table 1:** Baseline characteristics.

	Overall (*n* = 67)
AGE	
Mean (SD)	54.57 (11.58)
Median (Q1, Q3)	56 (48.5, 63)
Min–Max	25–74
GENDER	
F	13 (19.4%)
M	54 (80.6%)
BMI	
Mean (SD)	28.27 (3.42)
Median (Q1, Q3)	28.1 (25.8, 30.1)
Min–Max	22–42.5
ASA	
1	3 (4.5%)
2	42 (62.7%)
3	22 (32.8%)
AHI	
Mean (SD)	26.07 (9.4)
Median (Q1, Q3)	25.5 (21.68, 32.05)
Min–Max	5.5–47.1
Missing	3
Preop. SpO2	
Mean (SD)	82.75 (8.18)
Median (Q1, Q3)	83.5 (77, 88.25)
Min–Max	61–96
Missing	3
COMORBIDITY	
Diabetes	9 (13.4%)
Hypertension	25 (37.3%)
Cardiac diseases	11 (16.4%)
Tobacco smoking	15 (22.4%)
Preop. CPAP	20 (29.9%)
Severe OSA	
0	21 (31.3%)
1	46 (68.7%)
Severe OSA + soft palate and tongue base prolapse	
0	49 (73.1%)
1	18 (26.9%)
Severe OSA + soft palate prolapse	
0	54 (80.6%)
1	13 (19.4%)
TORS for tongue base resection	
1	67 (100.0%)
Anterolateral pharyngoplasty	
0	13 (19.4%)
1	54 (80.6%)
Tonsillectomy	
0	41 (61.2%)
1	26 (38.8%)
Anterior pharyngoplasty	
0	55 (82.1%)
1	12 (17.9%)
Barbed reposition pharyngoplasty	
0	62 (92.5%)
1	5 (7.5%)

Abbreviations: AHI, Apnea-Hypopnea Index; ASA, American Society of Anesthesiologists; CPAP, continuous positive airway pressure.

**Table 2:** Perioperative Course.

VARIABLE	
TORS DURATION (min)	
Mean (SD)	140.60 (38.36)
Median (Q1, Q3)	140 (120, 160)
Min–Max	60–275
EXTUBATION TIME (h)	
Mean (SD)	28.30 (15.11)
Median (Q1, Q3)	22 (20, 25)
Min–Max	0.00–72
AEC Use	24 (35.8%)
HFNC	21 (31.3%)
CONVENTIONAL	40 (59.7%)
ICU STAY (days)	
Mean (SD)	2.73 (1.77)
Median (Q1, Q3)	2 (1, 3)
Min–Max	1–9
HOSPITAL STAY (days)	
Mean (SD)	6.99 (3.02)
Median (Q1, Q3)	7 (5, 8)
Min–Max	3–22
SpO2 < 95 (episodes)	
0	45 (68.2%)
1	7 (10.6%)
2	5 (7.6%)
3	4 (6.1%)
4	2 (3%)
6	2 (3%)
7	1 (1.5%)
Missing	1
SpO2 < 92 (episodes)	
0	57 (87.7%)
1	5 (7.7%)
2	2 (3.1%)
4	1 (1.5%)
Missing	2
SpO2 < 95 continuous	
Mean (SD)	0.85 (1.61)
Median (Q1, Q3)	0.00 (0.00, 1)
Min–Max	0.00–7
Missing	1
SpO2 < 92 continuous	
Mean (SD)	0.20 (0.64)
Median (Q1, Q3)	0.00 (0.00, 0.00)
Min–Max	0.00–4
Missing	2

Abbreviations: TORS, transoral robotic surgery; ICU, intensive care unit; HNFC, high-flow nasal cannula; AEC, airway exchange catheter.

**Table 3:** Comparisons between groups.

	Conventional	HFNC	*p*
*n*	46	21	
AGE (mean ± SD)	55.3 (11.78)	52.95 (11.26)	0.445
GENDER (M; %)	37 (80.4)	17 (81)	1.000
BMI (mean ± SD)	28.61 (3.59)	27.53 (2.97)	0.236
ASA (*n*/%)			0.276
1	1 (2.2)	2 (9.5)	
2	28 (60.9)	14 (66.7)	
3	17 (37)	5 (23.8)	
AHI (mean ± SD)	25.66 (8.76)	26.99 (10.87)	0.603
Preop. SpO2 (mean ± SD)	83.32 (7.96)	81.50 (8.73)	0.414
Preop. CPAP (*n*/%)	13 (28.3)	7 (33.3)	0.894
Severe OSA	34 (73.9)	12 (57.1)	0.276
Severe OSA + soft palate and tongue base prolapse	13 (28.3)	5 (23.8)	0.933
Severe OSA + soft palate prolapse	5 (10.9)	8 (38.1)	0.023
TORS for tongue base resection	46 (100)	21 (100)	NA
Anterolateral pharyngoplasty	36 (78.3)	18 (85.7)	0.702
Tonsillectomy	22 (47.8)	4 (19)	0.049
Anterior pharyngoplasty	9 (19.6)	3 (14.3)	0.858
Barbed reposition pharyngoplasty	1 (2.2)	4 (19)	0.053
TORS DURATION (min) (mean ± SD)	136.3 (32.33)	150 (48.68)	0.177
EXTUBATION TIME (h) (mean ± SD)	31.09(17.24)	22.19 (5.31)	**0.024**
AEC (*n*/%)	6 (13)	18 (85.7)	**<0.001**
ICU STAY (days) (mean ± SD)	2.89 (1.78)	2.38 (1.75)	0.277
HOSPITAL STAY (days) (mean ± SD)	7.02 (3.15)	6.9 (2.77)	0.884
HYPERTENSION (*n*/%)	21 (45.7)	4 (19)	0.069
DIABETES (*n*/%)	7 (15.2)	2 (9.5)	0.804
CARDIAC DISEASE (*n*/%)	6 (13)	5 (23.8)	0.454
TOBACCO SMOKE (*n*/%)	7 (15.2)	8 (38.1)	0.077
SpO2 < 95 (episodes, *n*/%)			0.324
0	28 (62.2)	17 (81)	
1	5 (11.1)	2 (9.5)	
2	4 (8.9)	1 (4.8)	
3	4 (8.9)	0 (0.0)	
4	2 (4.4)	0 (0.0)	
5	2 (4.4)	0 (0.0)	
6	0 (0.0)	1 (4.8)	
SpO2 < 92 (episodes, *n*/%)			0.331
1	38 (86.4)	19 (90.5)	
2	4 (9.1)	1 (4.8)	
3	2 (4.5)	0 (0.0)	
4	0 (0.0)	1 (4.8)	
SpO2 < 95 continuous (mean ± SD)	1.00 (1.62)	0.52 (1.57)	0.266
SpO2 < 92 continuous (mean ± SD)	0.18 (0.5)	0.24 (0.89)	0.744

Abbreviations: ASA, American Society of Anesthesiologists; CPAP, continuous positive airway pressure; TORS, transoral robotic surgery; ICU, intensive care unit; HNFC, high-flow nasal cannula; AEC, airway exchange catheter.

## Data Availability

The datasets generated and analyzed in the present study are available upon reasonable request to the corresponding authors.
